# Assessing inpatient antibiotic use during COVID-19 surges with or without infectious diseases consultation

**DOI:** 10.1017/ash.2023.261

**Published:** 2023-09-29

**Authors:** Nicole Tommasi, Shira Doron, Gabriela Andujar-Vazquez, Maureen Campion

## Abstract

**Background:** Throughout the COVID-19 pandemic, increased inappropriate antibiotic use (AU) drove concern for antimicrobial resistance. Antimicrobial stewardship efforts are critical for combatting antimicrobial resistance. Our objective was to compare AU between SARS-CoV-2 delta and omicron variant surge periods in COVID-19 patients hospitalized at Tufts Medical Center (TMC) in Boston. Infectious diseases consultation (IDC) was mandatory for patients diagnosed with COVID-19 throughout the SARS-CoV-2 delta variant surge. During the SARS-CoV-2 omicron variant surge, IDC was optional for certain patient populations. Instead, the antibiotic stewardship program (ASP) reviewed these patients for appropriate medical management. We hypothesized that AU would increase during the SARS-CoV-2 omicron variant surge compared to the delta variant surge due to optional IDC because IDC would reduce inappropriate AU for suspected viral pneumonia. **Methods:** Retrospective medical record review of patients hospitalized with COVID-19 during the SARS-CoV-2 delta and omicron variant surges was conducted. We collected data regarding vital signs, white blood cell count (WBC), length of stay (LOS), steroid use, IDC, and AU (defined as percentage of patients receiving at least 1 antibiotic dose), with a separate category for antibiotics commonly used for bacterial pneumonia (ampicillin-sulbactam, azithromycin, cefepime, cefpodoxime, ceftazidime, ceftriaxone, doxycycline, piperacillin-tazobactam, vancomycin). We determined that 71 patients from each group were needed to detect an absolute difference of 20% in AU between surges with 75% power, based on the CDC estimate that 80% of patients hospitalized with COVID-19 receive an antibiotic. Unpaired *t* tests and χ^2^ analyses were conducted on demographic data. Inferential statistics assessed for differences between the 2 SARS-CoV-2 variant surges in AU and days of therapy (DOT), supplemental oxygen (SaO_2_), steroid use, and IDC utilizing a Wilcoxon rank-sum test and logistic regression analyses. **Results:** Results showed no significant differences in AU between surges (38.0% during the SARS-CoV-2 delta variant surge vs 42.3% during the SARS-CoV-2 omicron variant surge; *P* = .131). Disease severity was not different between surges as measured by steroid use, initial WBC, and SaO_2_. WBC was a predictor for AU in both surges (delta surge, *P* = 0.007; omicron surge, *P* = .002). Average LOS was higher throughout the SARS-CoV-2 delta variant surge for all patients (11.58 days during the delta surge, vs 5.97 days during the omicron variant surge; *P* = .047) and those who received antibiotics (18.44 days during the delta variant surge vs 6.70 days dring the omicron variant surge; *P* = .210). Total DOT was significantly longer during the SARS-CoV-2 delta variant surge for all antibiotics (463 DOT during the delta variant surge vs 277 DOT during the omicron variant surge; *P* = .047) and antibiotics commonly used for bacterial pneumonia (315 DOT during the delta variant surge vs 202 DOT during the omicron variant surge; *P* = .021). **Conclusions:** Making IDC optional for certain patient populations diagnosed with COVID-19 did not affect AU in a large, urban academic medical center with a comprehensive ASP.

**Disclosures:** None

